# Dietary Habits, Physical Activity and Body Mass Index in Transgender and Gender Diverse Adults in Italy: A Voluntary Sampling Observational Study

**DOI:** 10.3390/nu16183139

**Published:** 2024-09-17

**Authors:** Carmela Santangelo, Matteo Marconi, Angela Ruocco, Jiska Ristori, Stefania Bonadonna, Rosario Pivonello, Maria Cristina Meriggiola, Francesco Lombardo, Giovanna Motta, Chiara Michela Crespi, Maddalena Mosconi, Alessandro Oppo, Silvia Federici, Luca Bruno, Nunzia Verde, Alessandra Lami, Emanuela Bologna, Rosaria Varì, Maria Teresa Pagano, Luciana Giordani, Paola Matarrese, Flavia Chiarotti, Alessandra Daphne Fisher, Marina Pierdominici

**Affiliations:** 1 Reference Centre for Gender Medicine, Istituto Superiore di Sanità, 00161 Rome, Italy; carmela.santangelo@iss.it (C.S.); angela.ruocco@iss.it (A.R.); rosaria.vari@iss.it (R.V.); luciana.giordani@iss.it (L.G.); paola.matarrese@iss.it (P.M.); marina.pierdominici@iss.it (M.P.); 2 Andrology, Women’s Endocrinology and Gender Incongruence Unit, Florence University Hospital, 50100 Florence, Italy; jiska.ristori@unif.it (J.R.); alessandra.fisher@gmail.com (A.D.F.); 3 Istituto Auxologico Italiano, IRCCS, 20149 Milan, Italy; s.bonadonna@auxologico.it (S.B.); s.federici@auxologico.it (S.F.); 4 Dipartimento di Medicina Clinica e Chirurgia, Sezione di Endocrinologia, University Federico II, 80138 Naples, Italy; rosario.pivonello@unina.it (R.P.); lucabrun@hotmail.it (L.B.); nun.verde@gmail.com (N.V.); 5 Division of Gynecology and Physiopathology of Human Reproduction, Department of Medical and Surgical Sciences (DIMEC), IRCCS Azienda Ospedaliero-Universitaria di Bologna, S. Orsola Hospital, University of Bologna, 40138 Bologna, Italy; cristina.meriggiola@unibo.it (M.C.M.); alessandra.lami3@unibo.it (A.L.); 6 Laboratory of Semiology, Sperm Bank “Loredana Gandini”, Department of Experimental Medicine, Sapienza University of Rome, 00185 Rome, Italy; francesco.lombardo@uniroma1.it; 7 Division of Endocrinology, Diabetology and Metabolism, Azienda Ospedaliero-Universitaria Città della Salute e della Scienza di Torino, University of Turin, 10126 Turin, Italy; giovanna.motta.83@gmail.com (G.M.); ccrespi@cittadellasalute.to.it (C.M.C.); 8 Gender Identity Development Service, Hospital S. Camillo-Forlanini, 00152 Rome, Italy; mosconimadda@gmail.com; 9 Endocrinology Unit, Department of Medical Sciences and Public Health, University of Cagliari, 09124 Cagliari, Italy; aoppo@tiscali.it; 10 Istituto Nazionale di Statistica (ISTAT)-Dipartimento per la Produzione Statistica (DIPS), Direzione Centrale delle Statistiche Demografiche e del Censimento della Popolazione (DCDC), 00184 Rome, Italy; bologna@istat.it; 11 Department of Infectious Diseases, Istituto Superiore di Sanità, 00161 Rome, Italy; mariateresa.pagano@iss.it; 12 Independent Researcher, 00100 Rome, Italy; flavia.chiarotti@gmail.it

**Keywords:** transgender persons, gender diverse, diet, food, and nutrition, fruit and vegetables intake, red meat consumption, milk and yogurt consumption, fish consumption, body mass index, exercise

## Abstract

Transgender and gender-diverse (TGD) individuals continue to experience harassment and discrimination across various aspects of life, significantly impacting their physical and mental health. The scarcity of data on their general health, particularly regarding dietary habits, remains a challenge in developing effective healthcare strategies for this population. To address this gap, we analyzed selected dietary habits, physical activity (PA), and body mass index (BMI) among Italian TGD adults compared to the Italian general population (IGP). An online anonymous survey was conducted via the Computer Assisted Web Interviewing technique from June 2020 to June 2021. Participants were enrolled through clinical centers and TGD organizations. Data from 959 TGD adults were analyzed using chi-squared tests and logistic regression models. Key findings indicated that approximately 70% of TGD individuals consumed fewer servings of fruit and vegetables (FV) than recommended (five or more servings per day). Although red meat consumption was lower overall, a greater percentage of TGD individuals reported consuming more than three servings per week. Additionally, 58% of TGD participants indicated that they did not engage in any PA, compared to 36% of the IGP. Notably, significant differences in BMI were identified, with higher rates of overweight and obesity among TGD individuals assigned female at birth. These results underscore the urgent need for tailored nutritional guidelines and inclusive public health strategies to meet the specific health needs of the Italian TGD population. Expanding access to targeted interventions could contribute to improving overall well-being in this marginalized group.

## 1. Introduction

Poor nutrition remains a critical global issue, with far-reaching consequences for public health. The World Health Organization (WHO) reports that 30% of the global population is affected by some form of malnutrition [1]. Nutritional deficiencies have been strongly associated with chronic non-communicable diseases (NCDs), such as obesity, cardiovascular diseases, cancer, and mental health disorders, all of which have become increasingly prevalent in recent decades [2,3]. Conversely, a healthy diet, such as the Mediterranean diet and other plant-based diets, characterized by a high intake of fruit and vegetables (FV), lean proteins, and reduced meat consumption, is known to protect against NCDs when adhered to throughout life [4]. In this regard, WHO developed dietary guidelines that include recommendations on physical activity (PA) to prevent malnutrition in all its form [5]. In the same way, since 1979, Italy has developed Dietary Guidelines (IDGs) for Healthy Eating by the Research Center for Food and Nutrition of Council for Agricultural Research and Economics (CREA) [6], which are periodically revised and implemented [7].

Recent data regarding the eating behaviors of the Italian adult population during 2020–2021 showed an unhealthy lifestyle: less than 7% of adults had adequate intake of FV [8], and approximately 30% did not engage in any PA [9], leading to alarming levels of diet-related chronic diseases, with almost 43% of the adult population being overweight or obese [10].

Transgender and gender diverse (TGD) people represent a broad spectrum of individuals whose gender identities differ from the recorded sex at birth [11]. This population still faces harassment and discrimination in various aspects of life, including healthcare access, which significantly impacts both their physical and mental health. Furthermore, a lack of comprehensive information on their general health and health-related behaviors presents a major obstacle in developing effective healthcare strategies for this population [12,13]. An emerging body of research is exploring the relationships of TGD individuals with nutrition, eating disorders, and associated healthcare services. Available data suggest that this population is generally more likely to be diagnosed with an eating disorder or to engage in disordered eating behaviors than cisgender individuals [14,15]. This increased prevalence may be due, at least partially, to efforts to modify their body size or shape in alignment with their gender identity [16].

Unfortunately, to date, dietary recommendations worldwide are based on biological sex, not gender, and no dietary guidelines have been formulated for TGD people. The awareness of the importance of including gender identity in nutrition studies has only recently been acquired [17]. Available data indicate that women and men have different health beliefs that influence dietary behavior and food choices. Women tend to choose healthier food close to nutritional guidelines, while men prefer animal-derived foods [17,18]. A significant gap exists in research specifically focusing on the dietary habits and nutritional needs of TGD individuals, strongly suggesting the need for a comprehensive nutritional assessment of this population to better understand and manage their nutritional requirements [19,20,21]. To address this issue, we examined the dietary habits of the adult TGD population in Italy, focusing on the consumption of FV, red meat, fish, milk, and yogurt among individuals assigned female at birth (AFAB) and assigned male at birth (AMAB). We then compared these dietary patterns in AFAB and AMAB TGD individuals with those of their counterparts in the Italian General Population (IGP). Additionally, we analyzed PA and body mass index (BMI) to provide a more comprehensive understanding of overall health in this context. To our knowledge, this study is the first of its kind conducted in Italy and one of the few studies globally these important issues.

## 2. Materials and Methods

### 2.1. Participants and Procedure

The data collected for this study were part of an online anonymous survey conducted by Computer Assisted Web Interviewing (CAWI) technique from June 2020 to June 2021 to assess the health status of adult TGD people in Italy. Since this was the first survey on the health of the Italian TGD population, we included questions on various health-related topics covering sociodemographic characteristics, health behaviors such as dietary habits, experience in healthcare services, and adherence to cancer screenings. Details of the survey methodology have been published previously [22]. Briefly, participants were enrolled by the clinical centers and TGD organizations involved in the study, which directly invited their respective user bases. Recruitment was conducted through the distribution of flyers containing unique codes that provided access to the online platform. The inclusion criteria were defined as follows: self-identification as a TGD person, aged over 18 years, capable of reading and understanding Italian, residing in any of the 20 Italian regions, participating in the survey for the first time, and willing to provide written informed consent. ISS served as the coordinating center. The participating clinical centers were: (i) Florence University Hospital (Florence); (ii) University of Turin (Turin); (iii) University Federico II (Naples); (iv) University of Bologna (Bologna); (v) University of Cagliari (Cagliari); (vi) Hospital S. Camillo-Forlanini (Rome); (vii) Istituto Auxologico Italiano (Milan); (viii) Sapienza University of Rome (Rome). The Osservatorio Nazionale sull’Identità di Genere (ONIG)—National Observatory of Gender Identity and “The Bridge” Foundation also collaborated in the study. No incentives were offered for survey participation. For participants who did not complete the full survey, data were analyzed for all answered questions.

The Ethics Committee of the ISS approved this study (AOO-ISS 01/07/2019 0020061).

### 2.2. Health-Related Behaviours

In this study, we present the data obtained from the aforementioned survey regarding selected dietary habits, i.e., consumption of FV, red meat, fish, milk, and yogurt, as well as PA. As part of a comprehensive survey addressing various health-related topics, a limited set of questions regarding dietary habits was included. These specific parameters were selected due to their significant association with NCDs and cancer, both as risk factors and potential protective factors [2,3]. Height and weight were also determined to calculate BMI. Questions regarding FV, red meat, fish, milk, and yogurt consumption were derived from a validated quantitative Food Frequency Questionnaire (FFQ) [23]. Additionally, a short brief questionnaire [24] was administered to the participants to evaluate their PA level. The questions and corresponding response categories were formulated based on the Italian Dietary Guidelines (IDGs) [6] to ensure clear responses regarding the aforementioned parameters. Where necessary, we employed alternative response categories to streamline the interview process and improve the accuracy of subject classification.

#### 2.2.1. Fruit and Vegetables Consumption

Participants were asked to indicate how many servings of fruit and/or vegetables they consumed on a typical day. Response categories for FV consumption were: 2 or fewer servings per day, 3–4 servings per day, 5 or more servings per day.

According to IDGs [6], a minimum of 5 daily servings of FV is recommended for adults. A serving of vegetables is defined as a plate full of raw vegetables or half a plate of cooked vegetables, and a serving of fruit is defined as a medium fruit or 150 g of fruit.

#### 2.2.2. Fish and Red Meat Consumption

Fish intake was assessed by asking participants how many servings of fish, mollusks, or crustaceans they consumed per week. According to IDGs [6], a serving of fish is defined as: fresh/frozen fish approximately 150 g; preserved products (e.g., canned tuna, smoked salmon) approximately 50 g. The IDGs recommend 2–3 servings of fish per week.

Regarding red meat, participants were asked their intake of red meat (1 serving approximately 100 g) and/or cured meats (1 serving approximately 50 g) approximately per week. DGSs recommend meat consumption of no more than once a week. The response categories for both fish and red meat consumption were: 2 or less servings per week, 2–3 servings per week, more than 3 servings per week.

#### 2.2.3. Milk and Yogurt Intake

The participants were requested to indicate the number of servings of milk and/or yogurt they consumed daily. The response categories were as follows: no servings or one serving per day, 2 or more servings per day. According to IDGs [6] adult subjects should have a glass of milk (125 g) and/or a cup of yogurt (125 g) 3 times a day.

#### 2.2.4. Physical Activity

According to IDGs [6], adult subjects should do at least two and a half per week of moderate activity (e.g., brisk walking, housework, etc.), or one hour and a quarter of vigorous activity (e.g., swimming, jogging, cycling, dancing, etc.) or equivalent combinations of both, at least twice a week. The participants were requested to indicate the number of days per week on which they engaged in PA. The response categories were as follows: no PA; moderate PA (indicate the number of days per week), vigorous PA (indicate the number of days per week) [24].

### 2.3. Statistical Analysis

Quantitative data are presented as mean ± Standard Deviation (SD), median, first and third quartiles and range; qualitative data are synthesized by absolute frequencies and percentages.

Lifestyle variables concerning consumption of FV, red meat, fish, milk and yogurt, PA, and BMI, were compared between TGD population and IGP. First, we standardized the TGD population based on the age distribution of the IGP with the same sex assigned at birth (AMAB IGP for AMAB TGD, AFAB IGP for AFAB TGD) or the same gender identity (AFAB IGP for AMAB TGD, AMAB IGP for AFAB TGD). Next, within each age group (18–24.9 years, 25–34.9 years, 35–44.9 years, 45–54.9 years, ≥55 years), we calculated the observed and the expected frequencies for the levels of each lifestyle variable, using the frequency distribution of that variable observed in the TGD population and in the IGP population used for standardization. Data were sourced from the Istituto Nazionale di Statistica, ISTAT—National Institute of Statistics for the resident population as of 1 January 2021, within the age range of 18–69 years [25]. We then summed the standardized observed and expected frequencies across age groups to derive the standardized observed and expected frequencies in the overall groups of AMAB and AFAB TGD individuals, separately. Finally, we applied the chi-squared test to compare the standardized observed frequencies in AMAB and AFAB TGD individuals with the standardized expected frequencies (for details, see Table S1). We also compared lifestyle variable distributions between AMAB and AFAB individuals. To assess differences in the quantitative (age) and categorical (age classes, educational level, employment) variables describing AMAB and AFAB TGD individuals we used the Mann–Whitney U test (to account for potential non-normal distribution) and the Fisher’s exact probability test (appropriate also in case of low expected frequencies), respectively.

In order to assess differences in the lifestyle variables between AFAB and AMAB, accounting for age, education and occupation which are known to be associated with lifestyle [26,27,28], we applied logistic regression models (binomial or ordered, for categorical dependent variables with two or more than two levels, respectively), with each lifestyle variable as dependent variable in a separate model. “Sex assigned at birth” (AFAB vs. AMAB—reference level) was the explanatory variable, and “age” (continuous, expressed in years), “educational level” (secondary school/university (high level) vs. none/primary school/middle school (low level)—reference level), and employment status (homemaker/unoccupied/retired vs. occupied/student/other—reference level) were included as covariates.

Regression analyses were also repeated in AMAB and AFAB TGD groups separately, excluding the variable “sex assigned at birth” from the models. The overall significance of each logistic regression model was assessed using the likelihood ratio chi-square test comparing the model including only the constant (null model) to the model including the selected predictors (current model). The likelihood ratio chi-square (LR chi2) with degrees of freedom (df) and significance level were reported for each model (see Table S2). In addition, Odds Ratios (OR, for binary logistic models) or Prevalence Odds Ratios (POR, for ordered logistic models) with 95% Confidence Intervals (95%CIs) and significance levels were reported for each independent variable in the models.

All the analyses were conducted using STATA Statistical Software release 16 (StataCorp. 2019. Stata Statistical Software: Release 16. College Station, TX, USA: StataCorp LLC).

## 3. Results

This study included a cohort of 959 TGD participants, comprising 334 (34.8%) AMAB and 625 (65.2%) AFAB individuals. The mean age of the sample was 30 ± 11 years, with 71% of participants being under 35 years of age. A description of the sample in terms of age, educational level and employment status can be found in Table 1.

### 3.1. Fruit and Vegetables Consumption

**Figure 1:**
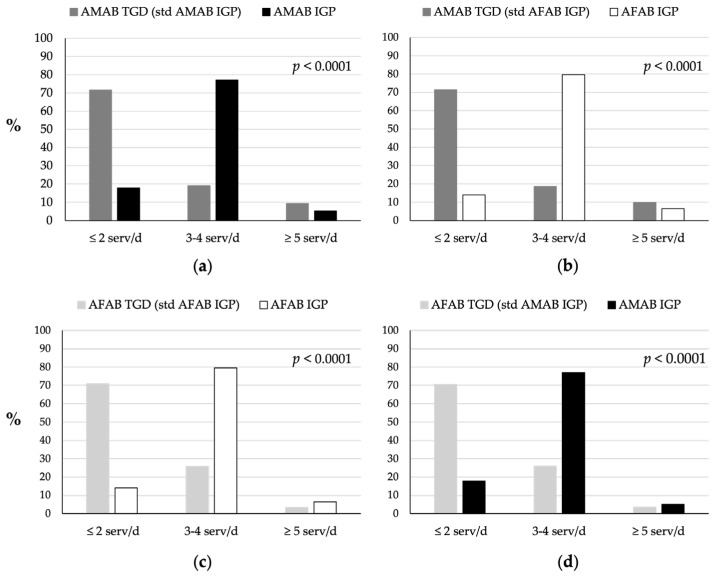
Comparison of fruit and vegetables consumption between TGD individuals and IGP. (**a**) Consumption patterns of AMAB TGD individuals compared to AMAB IGP individuals. (**b**) Consumption patterns of AMAB TGD individuals compared to AFAB IGP individuals. (**c**) Consumption patterns of AFAB TGD individuals compared to AFAB IGP individuals. (**d**) Consumption patterns of AFAB TGD individuals compared to AMAB IGP individuals. The TGD population was standardized based on the age distribution of the IGP of the same sex assigned at birth (AMAB IGP for AMAB TGD, AFAB IGP for AFAB TGD) or of the same gender identity (AFAB IGP for AMAB TGD, AMAB IGP for AFAB TGD). AFAB, assigned female at birth; AMAB, assigned male at birth; IGP, Italian general population; serv/d, servings per day; std, standardized; TGD, transgender and gender diverse people.

TGD individuals reported relatively low FV consumption (Figure 1). Approximately 70% of both AMAB and AFAB TGD individuals reported consuming two or fewer servings of FV per day, and approximately 20% reported consuming three to four servings per day, below the IDGs’ recommendation of at least five servings per day. Significant differences (*p* < 0.0001) in FV consumption were observed between TGD individuals and IGP, particularly when comparing AMAB TGD individuals with both AMAB and AFAB IGP (Figure 1a,b), and AFAB TGD individuals to both AFAB and AMAB IGP (Figure 1c,d). IGP individuals consumed more FV: only 18% of AMAB and 14% of AFAB IGP reported consuming fewer than two servings of FV per day, whereas 60–70% of both AMAB and AFAB IGP reported consuming three to four servings per day. However, the proportion of individuals consuming five or more servings of FV per day was comparable between TGD and IGP groups, averaging approximately 4–5%.

### 3.2. Red Meat Consumption

**Figure 2:**
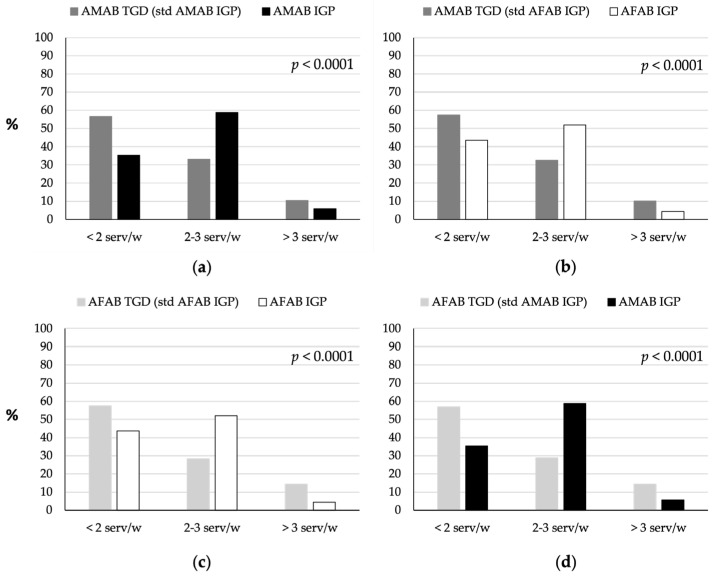
Comparison of red meat consumption between TGD individuals and IGP. (**a**) Red meat consumption patterns of AMAB TGD individuals compared to AMAB IGP individuals. (**b**) Red meat consumption patterns of AMAB TGD individuals compared to AFAB IGP individuals. (**c**) Red meat consumption patterns of AFAB TGD individuals compared to AFAB IGP individuals. (**d**) Red meat consumption patterns of AFAB TGD individuals compared to AMAB IGP individuals. The TGD population was standardized based on the age distribution of the IGP of the same sex assigned at birth (AMAB IGP for AMAB TGD, AFAB IGP for AFAB TGD) or of the same gender identity (AFAB IGP for AMAB TGD, AMAB IGP for AFAB TGD). AFAB, assigned female at birth; AMAB, assigned male at birth; IGP, Italian general population; serv/w, servings per week; std, standardized; TGD, transgender and gender diverse people.

TGD individuals generally consumed significantly less red meat than IGP (*p* < 0.0001; Figure 2), as shown by comparisons of AMAB TGD individuals with AMAB and AFAB IGP (Figure 2a,b), and AFAB TGD individuals with AFAB and AMAB IGP (Figure 2c,d). In detail, approximately 57% of both AMAB and AFAB TGD individuals consumed fewer than two servings per week, compared with approximately 35% of AMAB and 44% of AFAB IGP. Additionally, 33% of AMAB and 28% of AFAB TGD individuals consumed two to three servings per week, compared with 59% of AMAB and 52% of AFAB IGP. Conversely, the percentage of those consuming more than three servings per week was higher among AMAB (approximately 10%) and AFAB (approximately 14%) TGD individuals, compared with AMAB (approximately 6%) and AFAB IGP (approximately 4%).

### 3.3. Fish Consumption

**Figure 3:**
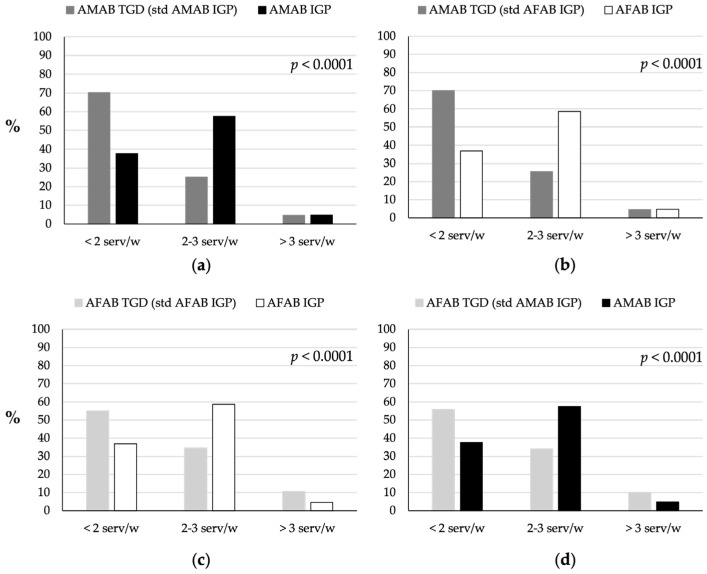
Comparison of fish consumption between TGD individuals and IGP. (**a**) Fish consumption patterns of AMAB TGD individuals compared to AMAB IGP individuals. (**b**) Fish consumption patterns of AMAB TGD individuals compared to AFAB IGP individuals. (**c**) Fish consumption patterns of AFAB TGD individuals compared to AFAB IGP individuals. (**d**) Fish consumption patterns of AFAB TGD individuals compared to AMAB IGP individuals. The TGD population was standardized based on the age distribution of the IGP of the same sex assigned at birth (AMAB IGP for AMAB TGD, AFAB IGP for AFAB TGD) or of the same gender identity (AFAB IGP for AMAB TGD, AMAB IGP for AFAB TGD). AFAB, assigned female at birth; AMAB, assigned male at birth; IGP, Italian general population; serv/w, servings per week; std, standardized; TGD, transgender and gender diverse people.

AMAB and AFAB TGD individuals consumed little fish, both in terms of the IDGs’ recommendation of at least two to three servings per week and compared to IGP (*p* < 0.0001; Figure 3). Approximately 70% of AMAB (Figure 3a,b) and 55% of AFAB (Figure 3c,d) TGD individuals consumed two or less servings per week, compared to approximately 37% of both AMAB and AFAB IGP. Additionally, approximately 25% of AMAB and 34% of AFAB TGD individuals consumed two to three servings per week, compared to approximately 57% and 58% of AMAB and AFAB IGP, respectively.

### 3.4. Milk and Yogurt Consumption

**Figure 4:**
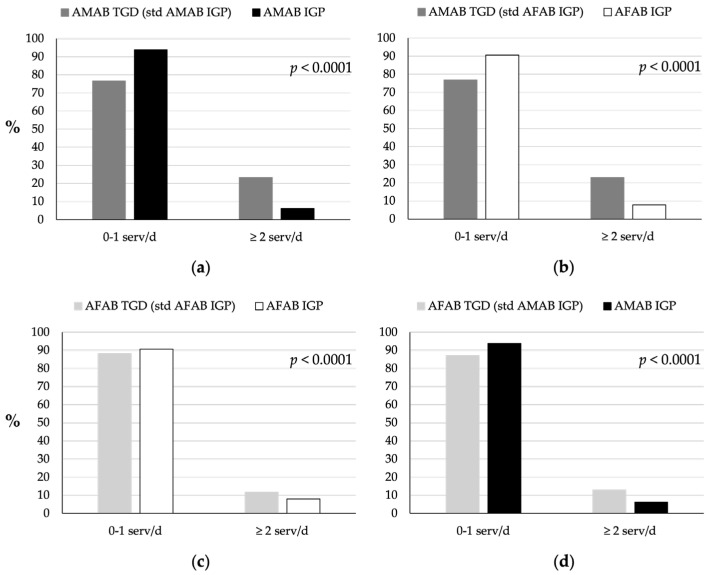
Comparison of milk and yogurt consumption between TGD individuals and IGP. (**a**) Milk and/or yogurt consumption patterns of AMAB TGD individuals compared to AMAB IGP individuals. (**b**) Milk and yogurt consumption patterns of AMAB TGD individuals compared to AFAB IGP individuals. (**c**) Milk and yogurt consumption patterns of AFAB TGD individuals compared to AFAB IGP individuals. (**d**) Milk and yogurt consumption patterns of AFAB TGD individuals compared to AMAB IGP individuals. The TGD population was standardized based on the age distribution of the IGP of the same sex assigned at birth (AMAB IGP for AMAB TGD, AFAB IGP for AFAB TGD) or of the same gender identity (AFAB IGP for AMAB TGD, AMAB IGP for AFAB TGD). AFAB, assigned female at birth; AMAB, assigned male at birth; IGP, Italian general population; serv/d, servings per day; std, standardized; TGD, transgender and gender diverse people.

TGD individuals consumed more milk and yogurt than IGP individuals (*p* < 0.0001; Figure 4). Approximately 77% of AMAB (Figure 4a,b) and 88% of AFAB TGD (Figure 4c,d) individuals consumed no servings or only one serving per day, compared to 94% and 92% of AMAB and AFAB IGP, respectively. Only 23% of AMAB TGD individuals and 12% of AFAB TGD individuals consumed the recommended dose of ≥2 servings per day; however, these percentages were higher than those observed in AMAB (6%) and AFAB (8%) IGP individuals.

### 3.5. Physical Activity

**Figure 5:**
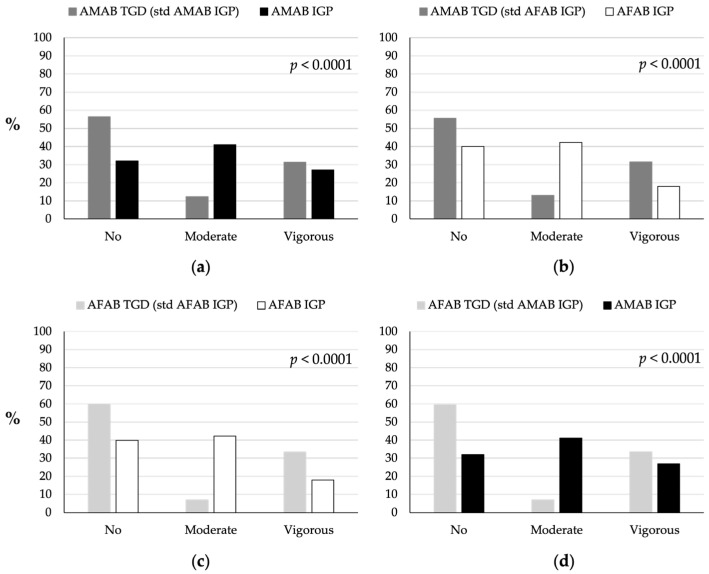
Comparison of physical activity levels between TGD individuals and IGP. (**a**) Physical activity patterns of AMAB TGD individuals compared to AMAB IGP individuals. (**b**) Physical activity patterns of AMAB TGD individuals compared to AFAB IGP individuals. (**c**) Physical activity patterns of AFAB TGD individuals compared to AFAB IGP individuals. (**d**) Physical activity patterns of AFAB TGD individuals compared to AMAB IGP individuals. The TGD population was standardized based on the age distribution of the IGP of the same sex assigned at birth (AMAB IGP for AMAB TGD, AFAB IGP for AFAB TGD) or of the same gender identity (AFAB IGP for AMAB TGD, AMAB IGP for AFAB TGD). AFAB, assigned female at birth; AMAB, assigned male at birth; IGP, Italian general population; std, standardized; TGD, transgender and gender diverse people.

A higher percentage of TGD individuals did not engage in any PA compared to IGP (56% of AMAB and 60% of AFAB TGD individuals vs. 32% of AMAB and 40% of AFAB IGP) (*p* < 0.0001; Figure 5). Fewer AMAB (12%) and AFAB (7%) TGD individuals engaged in moderate PA compared to AMAB (41%) and AFAB (42%) IGP. Conversely, both AMAB (31%) and AFAB (33%) TGD people participated in more intense PA than AMAB (27%) and AFAB (18%) IGP.

### 3.6. Body Mass Index

BMI was categorized as follows: underweight (BMI < 18.5), healthy weight (18.5–24.9), overweight (25–29.9), and obese (≥30) [29].

**Figure 6:**
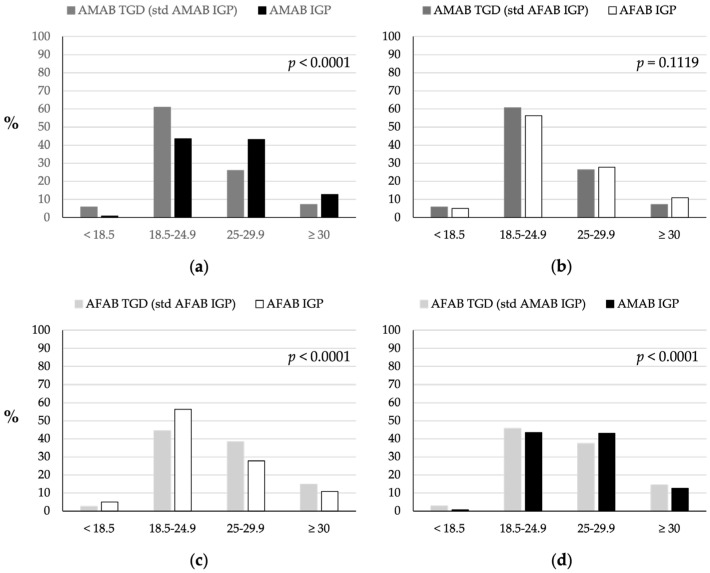
Comparison of BMI distribution between TGD individuals and IGP. (**a**) BMI distribution of AMAB TGD individuals compared to AMAB IGP individuals. (**b**) BMI distribution of AMAB TGD individuals compared to AFAB IGP individuals. (**c**) BMI distribution of AFAB TGD individuals compared to AFAB IGP individuals. (**d**) BMI distribution of AFAB TGD individuals compared to AMAB IGP individuals. The TGD population was standardized based on the age distribution of the IGP of the same sex assigned at birth (AMAB IGP for AMAB TGD, AFAB IGP for AFAB TGD) or of the same gender identity (AFAB IGP for AMAB TGD, AMAB IGP for AFAB TGD). AFAB, assigned female at birth; AMAB, assigned male at birth; IGP, Italian general population; std, standardized; TGD, transgender and gender diverse people.

Significant BMI differences were observed between TGD individuals and IGP, particularly when comparing AMAB TGD individuals to AMAB IGP, and AFAB TGD individuals to both AMAB and AFAB IGP (*p* < 0.0001; Figure 6). While 61% of the AMAB TGD population was of normal weight, compared to 43% of the AMAB IGP, a greater proportion of AMAB TGD individuals were underweight compared to AMAB IGP (6% vs. 0.8%). No statistically significant differences were identified between the AMAB TGD population and the AFAB IGP. A greater proportion of AFAB TGD individuals were found to be overweight and obese (38% and 15%, respectively) compared to the AFAB IGP (28% and 11%, respectively). These differences were less pronounced with the AMAB IGP (43% overweight and 13% obese). Notably, a smaller proportion of AFAB TGD individuals (2%) were underweight compared to the AFAB IGP (5%), but not compared to the AMAB IGP (0.8%).

### 3.7. Logistic Regression Analyses

The results are summarized in Table 2. Sex assigned at birth, age, educational level, and employment status significantly influenced TGD people lifestyles.

*FV optimal consumption*. Age was found to be a significant predictor of optimal FV consumption (five or more servings per week). In the overall TGD cohort, the OR for age was 1.028 (*p* = 0.033), indicating that with each additional year of age, the likelihood of achieving optimal FV intake increases by 2.8%. This association was even stronger in the AMAB TGD group, where the OR was 1.052 (*p* = 0.008), reflecting a 5.2% increase in the likelihood of optimal FV intake with each year of age.

*Read meat optimal consumption*. Age was identified as a significant predictor of optimal red meat consumption (no more than once a week). In the overall TGD group, the OR for age was 1.013 (*p* = 0.041), suggesting that with each additional year of age, the likelihood of meeting optimal red meat consumption guidelines increases by 1.3%. This relationship was stronger in the AFAB TGD group, where the OR was 1.029 (*p* = 0.001), reflecting a 2.9% increase in the likelihood of optimal consumption per year of age. However, this association was not significant in the AMAB TGD group, with an OR of 0.998 (*p* = 0.780), indicating no meaningful impact of age on red meat consumption in this subgroup. Employment status was also a significant predictor, but only in the AFAB TGD group, where the OR was 0.610 (*p* = 0.018). This suggests that employed AFAB individuals were 39% less likely to meet optimal red meat consumption guidelines compared to those who were not employed.

*Fish optimal consumption*. Age was a significant predictor of optimal fish consumption (two to three servings per week) in the AFAB TGD group, with an OR of 1.032 (*p* = 0.047). This indicates that for each additional year of age, the likelihood of optimal fish intake increases by 3.2% in this group. However, this relationship was not observed in the overall TGD cohort (OR = 1.016, *p* = 0.198) or the AMAB TGD group (OR = 1.000, *p* = 0.987). Additionally, higher education levels were significantly associated with optimal fish consumption. In the overall TGD cohort, individuals with higher education were 2.27 times more likely to achieve optimal fish intake (OR = 2.270, *p* = 0.049). This effect was even more pronounced in the AFAB TGD group, where higher education increased the likelihood of optimal fish consumption by over five times (OR = 5.042, *p* = 0.029).

*Milk and yogurt optimal consumption*. Higher education levels were associated with a lower likelihood of achieving optimal intake of milk and yogurt (three servings per day) in both the overall TGD cohort (OR = 0.667, *p* = 0.024) and the AMAB TGD group (OR = 0.484, *p* = 0.012). This suggests that individuals with higher education are less likely to meet the recommended intake in these groups. However, this association was not significant in the AFAB TGD group (OR = 0.793, *p* = 0.326). Additionally, unemployed AMAB TGD individuals were significantly less likely to achieve optimal milk and yogurt consumption, with an odds ratio of 0.527 (*p* = 0.029), indicating a 47.3% reduction in the likelihood of meeting the recommended intake compared to their employed counterparts.

*PA*. Age was identified as a significant predictor of PA participation in the AMAB TGD group, with an OR of 1.018 (*p* = 0.045), indicating that each additional year of age increased the likelihood of participating in PA by 1.8% in this group. Educational level was also a significant predictor of PA participation in both the overall TGD cohort (OR = 1.610, *p* = 0.003) and the AMAB TGD group (OR = 2.083, *p* = 0.005). This suggests that individuals with higher educational levels were more likely to engage in PA, with the effect being stronger in the AMAB group, where higher education increased the likelihood of PA participation by over two times. Employment status was a significant predictor of PA participation in the AFAB TGD group, with an OR of 0.617 (*p* = 0.018). This indicates that unemployed AFAB individuals were 38.3% less likely to participate in PA. However, employment status was not a significant predictor in the overall TGD cohort (OR = 0.790, *p* = 0.124) or the AMAB group (OR = 1.172, *p* = 0.511).

*BMI*. Sex assigned at birth was significantly associated with BMI, with AFAB TGD individuals exhibiting a notably higher likelihood of increased BMI compared to their AMAB counterparts. Specifically, AFAB individuals had a POR of 1.738 (*p* < 0.001), indicating a 73.8% higher likelihood of having a higher BMI. Age was also found to be a significant predictor of BMI, with older TGD individuals more likely to have a higher BMI (OR = 1.028, *p* < 0.001). This association was consistent across both AMAB and AFAB groups, showing that each additional year of age increased the likelihood of a higher BMI by 2.8%. Educational level significantly influenced BMI, with higher education levels associated with a reduced likelihood of having a higher BMI. In the overall TGD cohort, individuals with higher education had a 28.4% lower likelihood of having a higher BMI (POR = 0.716, *p* = 0.031). This effect was particularly notable in the AMAB group, where higher education was associated with a 40.1% lower likelihood of having a higher BMI (POR = 0.599, *p* = 0.051).

In summary, the findings highlight the significant influence of age, educational level, and employment status on various lifestyle behaviors among TGD individuals, with notable differences between AMAB and AFAB subgroups.

## 4. Discussion

In this study, we examined specific dietary habits, PA and BMI of the Italian adult TGD population through an interview survey focusing on key health determinants. The survey questions were designed in accordance with the IDGs [6]. Our findings indicated that most TGD individuals did not meet nutritional recommendations and led a sedentary lifestyle. Specifically, TGD individuals reported lower intake of FV and fish compared to the IGP. Despite a lower overall consumption of red meat, a higher percentage of TGD individuals consumed more than three servings per week. Conversely, TGD participants reported higher milk and yogurt consumption but were less likely to engage in PA. Gender identity, age, education level, and employment status emerged as key factors influencing these lifestyle behaviors. Older TGD individuals were more likely to meet dietary recommendations, while higher education levels were associated with healthier eating patterns and greater PA engagement. Interestingly, higher education was also linked to lower consumption of milk and yogurt. Additionally, AFAB TGD individuals were more likely to have higher BMI than their AMAB counterparts, with age and education also playing significant roles in BMI outcomes.

*FV consumption.* The World Health Report identified low intake of FV as one of the top ten global risk factors contributing to mortality [30]. Daily intake of FV protects against NCDs, with a strong association with cardiovascular benefits [31]. A diet poor in FV results in an insufficient intake of essential nutrients, such as vitamins, minerals and health promoting compounds like polyphenols and terpenoids [32,33,34]. Despite recommendation from most nutritional guidelines to consume at least five servings of FV per day [4,5,6], FV consumption remains low worldwide [35]. Limited research has explored FV consumption among the TGD population, with studies primarily conducted in the United States and New Zealand [36,37]. These studies, often focused on students, have yielded conflicting results. Among adults, Linsenmeyer et al. reported low FV intake among TGD men [38]. Our study expands on this by analyzing a larger sample of the adult TGD population. We found that TGD participants generally consumed fewer FV than the IGP, with a trend of increasing FV intake among AMAB TGD individuals as they age. This pattern of FV consumption may be influenced by gender roles, as seen in the general population where women tend to consume more FV, viewing them as healthier choices [17,39,40]. In our sample, most AMAB individuals identified as women [22], suggesting that their dietary behaviors might align with feminine norms. The increase in FV intake among older AMAB TGD individuals could reflect this alignment as they solidify their gender identity, indicating that gender roles may similarly shape dietary habits in the TGD community.

*Red Meat Consumption*. Gender roles influence dietary choices concerning red meat, with men consuming more red and processed meat than women [17,18]. This nutritional behavior, associated with traditional masculinity, is observed across various countries [41,42], even among young people [43]. In our study, TGD participants reported lower overall red meat consumption compared to the IGP group. However, a greater proportion of TGD individuals, particularly AFAB TGD, consumed more than three servings of red meat per week compared to their IGP counterpart. Given that most AFAB TGD participants identified as men [22], these findings align with previous research suggesting that red meat consumption among TGD individuals is often tied to expressions of strength and masculinity, potentially functioning as a way to affirm gender identity [40,43].

*Fish Consumption*. A higher frequency of fish intake is associated with healthier lifestyles due to its provision of high-quality proteins, essential vitamins, and omega-3 fatty acids, which offer protective effects against cardiovascular disease, cancer, and psychiatric disorders [44,45]. To date, to our knowledge, no studies have specifically examined fish consumption within the TGD population. Our study found that TGD participants consumed less fish than recommended, and significantly less compared to the IGP. Notably, age and education were positively correlated with better fish consumption, particularly among AFAB individuals. This pattern may be influenced by traditional gender roles, similar to trends observed in the consumption of FV and meat. In fact, existing research on the general population suggests that men typically consume more fish than women, possibly due to a preference for animal-derived foods [43]. However, other studies indicate that women’s increased awareness of the health benefits of fish may result in no significant differences in fish consumption between genders [45]. Moreover, the positive correlation between age, education, and improved fish consumption in our study reinforces the idea that older and more educated individuals may possess greater awareness of the health advantages of fish, leading to healthier dietary choices [46].

*Milk and yogurt consumption*. Dairy consumption, particularly the intake of milk and yogurt, has long been a subject of nutritional research due to its potential impact on human health and longevity. While milk and dairy products are widely recognized for their rich nutrient profiles including calcium, proteins, fats, minerals (phosphorus, magnesium, potassium, zinc, and selenium), and vitamins (vitamin A, riboflavin, and vitamin B-12), their relationship with overall mortality remains debated within the scientific community. Although some analyses report an increased risk of mortality with dairy products [47,48], most research supports the positive health effects of dairy, including a reduced risk of cardiovascular disease and overall mortality [49,50,51,52,53,54,55,56]. Based on this evidence, most dietary guidelines recommend the daily consumption of milk and dairy foods [5,6]. Research indicates significant differences in milk and yogurt consumption based on sex and gender [57,58], though there is no specific research on the milk and yogurt consumption patterns within the TGD population.

In our study, TGD individuals generally consumed more milk and yogurt compared to the IGP, although most did not meet the recommended intake. Higher education was associated with a reduced likelihood of reaching optimal consumption, particularly among AMAB TGD individuals. Additionally, unemployment negatively impacted milk and yogurt intake in this group. These results suggest that while TGD individuals may exhibit slightly better dietary patterns, there remains a significant gap in meeting recommended nutritional guidelines. This is particularly concerning given the elevated risk of osteoporosis in the TGD population [59,60]. The association between higher education levels and lower dairy intake is intriguing. Although education typically promotes healthier behaviors, as evidenced by higher fish consumption, this outcome highlights the complexity of nutritional knowledge and the varying perceptions of different food groups within more educated populations. Educated individuals may be more exposed to information about the potential risks of dairy consumption, such as increased mortality and lactose intolerance, leading to greater skepticism regarding its intake. Furthermore, they are more likely to explore alternative diets, such as plant-based or vegan options, which advocate for reduced dairy consumption, potentially explaining the lower yogurt intake despite its recognized health benefits [61]. Unemployment also emerged as a barrier to adequate dairy intake in our TGD population, indicating that public health strategies should not only focus on providing nutritional education but also address broader socioeconomic factors that may limit access to healthy food.

*PA*. Physical activity is a fundamental aspect of human health; regular PA plays an important role in weight management, reducing the risk of disease, and improving mental and social well-being [62]. Several factors influence PA levels, including sex, gender, age, and income [63]. Men engage in more PA than women across all age groups [64]. Men are more likely than women to associate a healthy lifestyle with regular PA rather than nutritional changes [65]. For TGD people, the literature indicates that they engage in PA less than cisgender individuals due to social, psychological, and physical factors [66]. This contributes to obesity, prevalent in approximately 40% of adult TGD, according to a 2020 United States community-based study [67]. In our study, we observed high rates of physical inactivity within the TGD cohort, particularly among younger individuals. However, a significant proportion of AMAB and AFAB TGD participants engaged in vigorous PA. Higher educational attainment was associated with greater PA levels. Our findings are consistent with previous research indicating elevated inactivity rates among TGD populations, likely due to barriers such as the lack of inclusive and safe sports facilities, as well as the risk of discrimination and bullying in sports and educational settings [68]. Additionally, challenges related to gender dysphoria could play a role [68]. The positive association between education and PA aligns with other observational studies, which suggest that higher educational attainment often correlates with better health literacy and higher levels of health-promoting behaviors in the general population [69]. However, many TGD individuals face barriers to education which may limit their ability to engage in healthier lifestyles [12,13]. Despite these barriers, some TGD individuals participated in intense PA, which may be motivated by factors such as body shape and self-acceptance, particularly among TGD men [70]. Similarly, TGD women may engage in exercise to increase muscle mass in areas like the gluteus, legs, or abdominals [71].

*BMI*. Our study found that a higher proportion of AMAB TGD individuals were of normal weight compared to AMAB IGP, but they were also more likely to be underweight. In contrast, a greater proportion of AFAB TGD individuals were overweight or obese compared to both AFAB and AMAB IGP. Age was strongly associated with BMI, as older TGD individuals were more likely to have a higher BMI. Additionally, higher educational levels were associated with a reduced likelihood of having a higher BMI. As previously noted, obesity is a common condition within the TGD population, particularly among AFAB individuals [67,72,73]. This may be attributed to various obesogenic factors (e.g., gender affirming hormonal treatment) that warrant further investigation in future studies that take the gender parameter into consideration. Moreover, our findings of a high percentage of underweight among AMAB TGD participants corroborate previous research that identified both underweight and obesity as significant concerns among TGD college students in the United States. This may be linked, at least in part, to body dissatisfaction within this population [74].

The findings presented have significant implications for both practical applications and public health initiatives targeting the TGD population. The observed dietary behaviors among TGD individuals, such as low consumption of FV and fish, suggest potential long-term health risks, particularly concerning cardiovascular diseases and cancer. Notably, TGD individuals face an elevated risk of chronic conditions, such as cardiovascular diseases, and experience higher mortality rates from these conditions compared to the cisgender population [75,76,77]. Gender identity, age, education level, and employment status influence dietary habits, highlighting that the TGD population is not homogeneous. Therefore, health policies must be adaptable and responsive to the diverse experiences and needs of various TGD subgroups.

Several public health strategies can be implemented. For example, culturally competent health campaigns promoting optimal nutrition should consider gender identity and perceptions of food choices to ensure that messages resonate with TGD individuals and are inclusive of their experiences. Additionally, increasing research and data collection is crucial. Our study highlights a significant gap in research on dietary habits within the TGD population, emphasizing the need for further investigation into how factors such as hormone therapy, gender identity, and lifestyle influence eating behaviors. It would be beneficial for policymakers to prioritize funding for research that explores these relationships and supports the development of evidence-based dietary guidelines that address the unique health needs of the TGD community. Healthcare providers would benefit from training in culturally competent care for TGD individuals, including guidance on addressing nutritional needs and promoting healthy dietary practices. This training should emphasize the importance of optimal nutrition and provide strategies for effectively communicating these recommendations to TGD patients.

The importance of developing public health policies and programs sensitive to the demographic variables and specific factors influencing the well-being of TGD individuals is further supported by our data on other lifestyle parameters, such as PA and BMI. Public health interventions should focus on creating inclusive and safe environments for PA. These initiatives could include the development of TGD-friendly sports programs and facilities, anti-bullying policies in educational settings, and mental health support to address barriers related to gender dysphoria [66].

The significant association between age and BMI across both AMAB and AFAB TGD subgroups further underscores the cumulative impact of time on body weight, suggesting that aging within this population may exacerbate weight-related health disparities. Weight management interventions should consider the role of aging, especially as the TGD population continues to grow older and requires more tailored healthcare services. Moreover, the influence of education on BMI, particularly among AMAB TGD participants, points to broader social determinants of health intersecting with gender identity. Tailored weight management programs that account for the specific needs of TGD individuals, such as the effects of hormone therapy on body composition and the psychological aspects of body image, are crucial.

The results of this study should be interpreted considering some limitations. Firstly, the brief questionnaire, used to evaluate adherence to the dietary recommendations of the IDGs, was part of a more comprehensive tool designed to assess a wide range of health-related topics in the adult TGD population in Italy [22]. While this broad approach allowed for a general overview, it necessitated a reduction in the number of questions for each specific area. For instance, while understanding the consumption of non-alcoholic beverages could have yielded valuable insights into dietary patterns and their impact on BMI, the breadth of the survey limited our ability to explore these aspects in detail. This compromise may have resulted in an incomplete understanding of certain lifestyle factors. Another critical limitation is the reliance on self-reported data, which introduces the risk of bias due to inaccurate reporting. Participants may have underreported or overreported their behaviors, either intentionally or unintentionally. For example, participants might have misreported their dietary intake or PA levels, skewing the results and possibly masking the true associations between lifestyle factors and health outcomes in the Italian TGD population. Moreover, the small number of nonbinary individuals (less than 10%) in the study population [22] limited our ability to disaggregate the data by gender identity. Consequently, the analysis was conducted based on sex assigned at birth, which may not fully capture the health experiences of nonbinary participants. Additionally, the absence of gender identity measures in national data collections, such as population censuses and medical records, hindered our ability to compare the study sample to the cisgender population. It is also important to address the non-significant findings in our study. For example, the lack of association between age and red meat consumption in the AMAB TGD group and the non-significant relationship between education and optimal milk and yogurt intake in the AFAB TGD group suggest that other factors, such as cultural influences, dietary preferences, or access to resources, may play a more prominent role in influencing these behaviors within these subgroups.

Finally, our study was focused on the adult population residing in Italy, and participants were required to read and understand Italian. This language requirement may have excluded non-Italian-speaking TGD individuals living in Italy, leading to a potential underrepresentation of certain groups in the study. As a result, the findings may not fully reflect the diversity of experiences within the Italian TGD population.

To address these limitations, future research should incorporate more comprehensive approaches to data collection that facilitate a deeper exploration of lifestyle factors and their intersections with variables such as educational background, socioeconomic status, and pre-existing health conditions. Additionally, larger and more diverse samples, particularly with greater representation of nonbinary individuals, will be crucial to better understand gender-related differences in health outcomes.

Finally, efforts to improve data accuracy, such as combining self-reported data with objective measures, should also be prioritized. Future research should also focus on longitudinal studies to understand the long-term effects of dietary and PA habits on the health outcomes of TGD individuals.

Despite these limitations, the study exhibits some notable strengths. The large sample size of TGD participants enabled us to substantiate existing evidence on TGD lifestyles effectively. Moreover, this study introduced new dietary data, which, when integrated with previous findings, could serve as a critical foundation for future research and public health interventions. A key strength of the study lies in its collaborative nature, bringing together institutions, clinical centers, and community organizations. Continued partnerships with these entities may enhance recruitment strategies, improve the cultural relevance of interventions, and ensure that research findings are successfully translated into practice. Engaging with community stakeholders will be crucial for addressing the unique needs of the TGD population, thereby ensuring that future research efforts contribute to meaningful improvements in health outcomes.

## 5. Conclusions

Our findings indicate that TGD individuals face significant challenges in adhering to dietary and PA guidelines, which may contribute to long-term health risks. Age, gender identity, education level, and employment status emerged as influential factors in shaping these behaviors, underscoring the need for tailored health interventions. This research contributes to a growing body of literature, providing critical insights into the health disparities experienced by TGD individuals and underscoring the importance of inclusive and culturally competent public health strategies. Future research should expand on these findings, focusing on diverse and representative samples, longitudinal data, and partnerships with community organizations to develop targeted interventions that address the specific health needs of this population.

## Figures and Tables

**Table 1:** Sociodemographic characteristics of AMAB and AFAB TGD individuals.

	TGD Population	AMAB TGD	AFAB TGD	
				***p, *** **AMAB TGD** vs. AFAB TGD *
**Age**				**<0.001**
N. of respondents	959	334	625	
Mean (SD)	30.46 (11.13)	34.46 (12.80)	28.32 (9.46)	
Median (Q1, Q3)	26 (22, 37)	31 (23, 45)	25 (21, 33)	
Min, Max	18, 68	18, 68	18, 61	
	**n (%)**	**n (%)**	**n (%)**	***p, *** **AMAB TGD** vs. AFAB TGD **
**Age group**				**<0.001**
18–24.9	396 (41.29)	105 (31.44)	291 (46.56)	
25–34.9	286 (29.82)	84 (25.15)	202 (32.32)	
35–44.9	138 (14.39)	57 (17.07)	81 (12.96)	
45–54.9	98 (10.22)	58 (17.37)	40 (6.40)	
≥55	41 (4.27)	30 (8.99)	11 (1.76)	
Total	959 (100)	334 (100)	625 (100)	
**Educational level**				**0.001**
Low	224 (23.48)	97 (29.31)	127 (20.39)	
High	730 (76.52)	234 (70.69)	496 (79.61)	
Total	954 (100)	331 (100)	623 (100)	
**Employment**				**<0.001**
**No**	257 (26.97)	128 (38.79)	129 (20.71)	
**Yes**	696 (73.03)	202 (61.21)	494 (79.29)	
**Total**	953 (100)	330 (100)	623 (100)	

Significant *p* values are shown in bold. * Mann–Whitney *p*; ** Fisher’s *p*. AFAB, assigned female at birth; AMAB, assigned male at birth; TGD, transgender and gender diverse people.

**Table 2:** Logistic regression analyses in the total TGD population, AMAB and AFAB TGD individuals.

	TGD Population		AMAB TGD		AFAB TGD	
	OR (95% CIs)	*p*	OR (95% CIs)	*p*	OR (95% CIs)	*p*
**Fruit and vegetables optimal consumption** (Yes vs. No)						
**Sex assigned at birth** (AFAB vs. AMAB)	1.116 (0.575–2.167)	0.745				
**Age** (x + 1 vs. x)	1.028 (1.002–1.054)	**0.033**	1.052 (1.013–1.092)	**0.008**	1.006 (0.969–1.045)	0.742
**Educational level** (High vs. Low)	2.107 (0.868–5.118)	0.100	3.013 (0.631–14.399)	0.167	1.679 (0.571–4.940)	0.347
**Employment** (No vs. Yes)	0.697 (0.325–1.496)	0.354	0.935 (0.299–2.922)	0.908	0.590 (0.201–1.734)	0.338
**Red meat optimal consumption** (Yes vs. No)						
**Sex assigned at birth** (AFAB vs. AMAB)	0.771 (0.580–1.026)	0.075				
**Age** (x + 1 vs. x)	1.013 (1.001–1.025)	**0.041**	0.998 (0.981–1.015)	0.780	1.029 (1.012–1.047)	**0.001**
**Educational level** (High vs. Low)	1.094 (0.802–1.493)	0.571	1.072 (0.653–1.759)	0.784	1.184 (0.787–1.781)	0.418
**Employment** (No vs. Yes)	0.865 (0.639–1.170)	0.346	1.293 (0.812–2.060)	0.279	0.610 (0.405–0.920)	0.018
**Fish optimal consumption** (Yes vs. No)						
**Sex assigned at birth** (AFAB vs. AMAB)	0.939 (0.522–1.688)	0.832				
**Age** (x + 1 vs. x)	1.016 (0.992–1.040)	0.198	1.000 (0.965–1.035)	0.987	1.032 (1.000–1.065)	**0.047**
**Educational level** (High vs. Low)	2.270 (1.003–5.138)	**0.049**	1.220 (0.421–3.537)	0.714	5.042 (1.184–21.473)	**0.029**
**Employment** (No vs. Yes)	0.676 (0.339–1.348)	0.266	0.652 (0.239–1.773)	0.402	0.632 (0.239–1.673)	0.355
**Milk and Yogurt optimal consumption** (Yes vs. No)						
**Sex assigned at birth** (AFAB vs. AMAB)	0.886 (0.633–1.240)	0.480				
**Age** (x + 1 vs. x)	0.995 (0.981–1.010)	0.512	1.002 (0.982–1.022)	0.861	0.989 (0.968–1.010)	0.291
**Educational level** (High vs. Low)	0.667 (0.468–0.949)	**0.024**	0.484 (0.275–0.855)	**0.012**	0.793 (0.500–1.259)	0.326
**Employment** (No vs. Yes)	0.739 (0.512–1.067)	0.107	0.527 (0.296–0.936)	**0.029**	0.937 (0.582–1.510)	0.790
**Physical activity** (Yes vs. No)						
**Sex assigned at birth** (AFAB vs. AMAB)	1.193 (0.897–1.586)	0.226				
**Age** (x + 1 vs. x)	1.004 (0.992–1.016)	0.568	1.018 (1.000–1.036)	**0.045**	0.991 (0.974–1.007)	0.269
**Educational level** (High vs. Low)	1.610 (1.179–2.199)	**0.003**	2.083 (1.247–3.479)	**0.005**	1.373 (0.921–2.048)	0.120
**Employment** (No vs. Yes)	0.790 (0.584–1.067)	0.124	1.172 (0.731–1.878)	0.511	0.617 (0.414–0.919)	**0.018**
	**POR (95% CIs)**	***p***	**POR (95% CIs)**	***p***	**POR (95% CIs)**	***p***
**Body Mass Index** (4 classes)						
**Sex assigned at birth** (AFAB vs. AMAB)	1.738 (1.308–2.309)	**<0.001**				
**Age** (x + 1 vs. x)	1.028 (1.017–1.041)	**<0.001**	1.028 (1.010–1.046)	**0.002**	1.029 (1.013–1.046)	**<0.001**
**Educational level** (High vs. Low)	0.716 (0.528–0.970)	**0.031**	0.599 (0.358–1.003)	0.051	0.779 (0.533–1.140)	0.199
**Employment** (No vs. Yes)	1.174 (0.870–1.584)	0.293	0.933 (0.575–1.513)	0.778	1.342 (0.913–1.972)	0.135

Significant *p* values are shown in bold. 95% CIs, 95% confidence intervals; AFAB, assigned female at birth; AMAB, assigned male at birth; OR, odds ratio; POR prevalence odds ratio; TGD, transgender and gender diverse people.

## Data Availability

De-identified data may be shared and made available upon reasonable request to the corresponding author and subject to an approved proposal and data access agreement due to ethical reasons.

## Supplementary Materials

The following supporting information can be downloaded at: https://www.mdpi.com/article/10.3390/nu16183139/s1, Table S1: Example of standardization, AMAB TGD vs. AMAB IGP, Fruit and vegetable consumption; Table S2: Overall significance of each logistic regression model used.
