# Supplementary material for: Dietary Habits, Physical Activity and Body Mass Index in Transgender and Gender Diverse Adults in Italy: A Voluntary Sampling Observational Study
Source: Nutrients. 2024 Sep 17;16(18):3139. doi: 10.3390/nu16183139 (PMC11435112; doi:10.3390/nu16183139)
Supplement: Supplementary file 1 [file nutrients-16-03139-s001.zip › Table S1 .docx]

**Table S1.** Example of standardization, AMAB TGD vs AMAB IGP, Fruit and vegetable consumption

AMAB TGD observed absolute (Panel A) and percent (Panel B) frequencies

| Panel A |  |  |  |  |  | Panel B |  |  |  |  |
| --- | --- | --- | --- | --- | --- | --- | --- | --- | --- | --- |
| **age (yrs)** | **<=2 serv/d** | **3-4 serv/d** | **>=5 serv/d** | ***Tot*** |  | **age (yrs)** | **<=2 serv/d** | **3-4 serv/d** | **>=5 serv/d** | ***Tot*** |
| **18-24** | 85 | 20 | 3 | *108* |  | **18-24** | 78.7 | 18.5 | 2.8 | *100.0* |
| **25-34** | 63 | 18 | 3 | *84* |  | **25-34** | 75.0 | 21.4 | 3.6 | *100.0* |
| **35-44** | 44 | 14 | 0 | *58* |  | **35-44** | 75.9 | 24.1 | 0.0 | *100.0* |
| **45-54** | 37 | 16 | 5 | *58* |  | **45-54** | 63.8 | 27.6 | 8.6 | *100.0* |
| **55+** | 22 | 4 | 5 | *31* |  | **55+** | 71.0 | 12.9 | 16.1 | *100.0* |
| ***Tot*** | *251* | *72* | *16* | *339* |  | ***Tot*** | *74.0* | *21.2* | *4.7* | *100.0* |

AMAB IGP observed absolute (Panel A) and percent (Panel B) frequencies

| Panel A |  |  |  |  |  | Panel B |  |  |  |  |
| --- | --- | --- | --- | --- | --- | --- | --- | --- | --- | --- |
| **age (yrs)** |  |  |  | ***Tot*** |  | **age (yrs)** | **<=2 serv/d** | **3-4 serv/d** | **>=5 serv/d** | ***Tot*** |
| **18-24** |  |  |  | *2,141,632* |  | **18-24** | 22.8 | 71.6 | 5.6 | *100.0* |
| **25-34** |  |  |  | *3,200,152* |  | **25-34** | 19.8 | 75.1 | 5.1 | *100.0* |
| **35-44** |  |  |  | *3,749,031* |  | **35-44** | 19.7 | 75.9 | 4.4 | *100.0* |
| **45-54** |  |  |  | *4,706,498* |  | **45-54** | 20.3 | 74.6 | 5.1 | *100.0* |
| **55+** |  |  |  | *10,255,451* |  | **55 e +** | 14.8 | 79.8 | 5.4 | *100.0* |
| ***Tot*** |  |  |  | *24,052,764* |  | ***Tot*** | *17.8* | *77.1* | *5.2* | *100.0* |

AMAB TGD standardized observed absolute (Panel A) and percent (Panel B) frequencies

| Panel A |  |  |  |  |  | Panel B |  |  |  |  |
| --- | --- | --- | --- | --- | --- | --- | --- | --- | --- | --- |
| **age (yrs)** | **<=2 serv/d** | **3-4 serv/d** | **>=5 serv/d** | ***Tot*** |  | **age (yrs)** | **<=2 serv/d** | **3-4 serv/d** | **>=5 serv/d** | ***Tot*** |
| **18-24** | 24 | 6 | 1 | *30* |  | **18-24** | 78.7 | 18.5 | 2.8 | *100.0* |
| **25-34** | 34 | 10 | 2 | *45* |  | **25-34** | 75.0 | 21.4 | 3.6 | *100.0* |
| **35-44** | 40 | 13 | 0 | *53* |  | **35-44** | 75.9 | 24.1 | 0.0 | *100.0* |
| **45-54** | 42 | 18 | 6 | *66* |  | **45-54** | 63.8 | 27.6 | 8.6 | *100.0* |
| **55+** | 103 | 19 | 23 | *145* |  | **55+** | 71.0 | 12.9 | 16.1 | *100.0* |
| ***Tot STD*** | *243* | *65* | *31* | *339* |  | ***Tot*** | *71.6* | *19.2* | *9.3* | *100.0* |

AFAB, assigned female at birth; AMAB, assigned male at birth; IGP, Italian general population; TGD, transgender and gender diverse people; serv/d, servings per day; STD, standardized; yrs, years.
