# Supplementary material for: Dietary Habits, Physical Activity and Body Mass Index in Transgender and Gender Diverse Adults in Italy: A Voluntary Sampling Observational Study
Source: Nutrients. 2024 Sep 17;16(18):3139. doi: 10.3390/nu16183139 (PMC11435112; doi:10.3390/nu16183139)
Supplement: Supplementary file 1 [file nutrients-16-03139-s001.zip › Table S2.docx]

**Table S2**. Overall significance of each logistic regression model used

|  | **TGD population** | | **AMAB TGD** | | **AFAB TGD** | |
| --- | --- | --- | --- | --- | --- | --- |
|  | **LR chi^2^ (df)** | ***p*** | **LR chi^2^ (df)** | ***p*** | **LR chi^2^ (df)** | ***p*** |
| **Fruit and vegetables optimal consumption** *(Yes vs No)* | 8.96 (4) | *0.062* | 9.87 (3) | ***0.020*** | 2.38 (3) | *0.497* |
| **Red meat**  **optimal consumption** *(Yes vs No)* | 10.20 (4) | ***0.037*** | 1.27 (3) | *0.736* | 16.85 (3) | ***<0.001*** |
| **Fish**  **optimal consumption** *(Yes vs No)* | 8.63 (4) | *0.071* | 1.09 (3) | *0.780* | 11.97 (3) | ***0.008*** |
| **Milk and Yogurt**  **optimal consumption** *(Yes vs No)* | 7.07 (4) | *0.132* | 8.90 (3) | ***0.031*** | 2.00 (3) | *0.573* |
| **Physical activity**  *(Yes vs No)* | 16.94 (4) | ***0.002*** | 12.34 (3) | ***0.006*** | 11.33 (3) | ***0.002*** |
| **Body Mass Index**  *(4 classes)* | 35.81 (4) | ***<0.001*** | 13.25 (3) | ***0.004*** | 18.87 (3) | ***<0.001*** |

Significant p values are shown in bold. LR chi^2^, likelihood ratio chi-square; df, degrees of freedom; AFAB, assigned female at birth; AMAB, assigned male at birth; TGD, transgender and gender diverse people.
